# Supplementary material for: Perceived coercion, perceived pressures and procedural justice arising from global lockdowns during the COVID-19 pandemic: A scoping review
Source: PLOS Glob Public Health. 2023 Mar 16;3(3):e0001250. doi: 10.1371/journal.pgph.0001250 (PMC10019622; doi:10.1371/journal.pgph.0001250)
Supplement: Attachment — Submitted filename: SR_C19_Response to Reviewers.docx [file pgph.0001250.s002.docx]

**Response to Reviewers**

| **Comment by reviewer** | **Change to manuscript** |
| --- | --- |
| *Reviewer One* |  |
| Abstract  Whether perceived coercion, pressure, and procedural injustice were reported by medical professionals or by the general public is not clear from the abstract.  Arksey & O’Malley’s (2005) framework for conducting scoping reviews” The sentence seems incomplete; can include was used | We thank the reviewer for this comment. The abstract has now been amended to reflect that the scoping review refers to the general population’s experience. We have also amended the sentence referring to Arksey & O’Malley’s framework. |
| Introduction  Few sentences are difficult to understand (highlighted in the manuscript). The introduction discusses perceived coercion in relation to admissions to mental health facilities. The rationale involves perspective of the general population. | We cannot see the highlighted changes to the text but have amended the introduction to more clearly show the parallels between mental health detentions and lockdown, both of whom involve restrictions to freedom of movement and may give rise to perceived coercion depending on whether the individual perceives their circumstances as within their control or of their choice. |
| Materials and Methods  Details regarding methods followed while implementing Arksey & O’Malley’s (2005) framework can be provided. Search Strategy: Other search terms were also tested but excluded because of the limited relevance of the resulting studies: It's better to share few words/phrases that considered but were excluded. This will help the reader. | We agree with the reviewer’s comment regarding search terms and have included the following examples within the article:  “Examples of excluded search terms were: (adherence OR acceptance OR agreement OR trust OR distrust OR compliance OR willing*) OR (perceived coerc* OR percept* coerc* OR pressure OR force OR influence OR control OR threat OR justice) AND (quarantine OR lockdown OR isolation), as many of the search results were non-specific to the COVID-19 pandemic; and (perceived coerc* OR percept* coerc* OR pressure OR force OR influence OR control OR threat OR justice) AND (COVID-19 or coronavirus), as most results were in relation to mental health admissions.”  A scoping review aims to map what is known within the literature about a topic using a range of different sources. For the purpose of this review, we included only published articles. This process consisted of identifying a clear research question; searching for and selecting relevant studies that aimed to answer our research question; and charting, summarising and reporting the findings of these studies. The framework does not include a quality assessment of included articles, as scoping reviews are designed to be rapid and broad in nature, as well as inclusive of all types of articles. Unlke systematic reviews, they are not intended primarily to judge research quality or identify sources of bias. |
| Results  There is some inconsistency in the numbers presented. In the PRISMA chart (41628-41377), it should be 251. But chart shows 252. The full text screening section needs to list the articles that were included and how many articles were excluded. Also, i.e. exclusion should be 211 articles (251-211=40) but not clear. The number of studies is 39 in the text above, but the table lists 40 studies.   Also, Out of 40 included 31 were primary articles. The details regarding remaining 9 articles can be provided. The data extraction pattern is separate for the review paper and policy documents. Details are needed on how the findings from the commentaries, reviews, policy documents, and letters differed from the original articles | Thank you – we have made those changes to reflect that the review included 40 studies. Data regarding the remaining articles have also been added.  The data extraction pattern itself did not differ between articles. All and any information pertaining to perceived coercion, pressures or procedural justice or related constructs was extracted from the included articles. However, the nature of the data differed between these. The original articles (and letter) tended to present a statistical finding, the review presented one key finding linked to perceived coercion and pressures (i.e. entrapment), the policy documents made reference to difficulties in ensuring procedural justice in relation to lockdown and the commentaries provided opinions or anecdotal evidence linked to perceived coercion, pressures or procedural justice. |
| Discussion  Expand the abbreviation on first use IAPT | Thank you – we have now amended this. |
| *Reviewer Two* |  |
| 1. Firstly, the manuscript is not suited to a public health journal in its present form, as it is largely raising questions around ethics and politics, and the acceptability of lockdowns, rather than the implications for global health ethics or health access or the impact of the lockdown or perceptions around lockdowns or distrust on health access or the functioning of the health system. My suggestion for the authors would be to include aspects of either global health access or ethics, to make it more relevant for publication in the journal. | We thank the reviewer for this comment, however, kindly disagree. The topic of perceived coercion is at the very heart of ethics. Ethical practice and policy is founded upon nonmaleficence, beneficence, justice, and respect for others’ autonomy. Perceived coercion occurs when an individual perceives their circumstances as arising from decisions made by others with authority that are viewed by them as non-beneficent, maleficent and unjust. Restrictions to autonomy and freedom of movement can give rise to higher levels of perceived coercion, as evidenced by the extensive literature pertaining to mental health admissions. However, it is perceived coercion – rather than documented coercion – that shows poorer prognostic outcomes within a psychiatric context, as some of those who are detained do not perceive their circumstances as coercive whilst some who are admitted voluntarily feel coerced.  Lockdown shares commonalities with mental health admissions due to the physical restrictions experienced by individuals. One of the focal aims of this review was to apply the construct of perceived coercion to lockdown, in order to begin to chart what the outcomes of people across the globe may be in the future. As psychologists and ethicists, we are interested in understanding how individuals’ perceptions may influence their emotional wellbeing. As the ethical implications of lockdown affect many countries worldwide, it becomes a global public and mental health issue.  We agree with the reviewer that the review does not examine health access or the functioning of health systems as those areas are beyond the remit of the review. However, most of the included articles specifically speak to the acceptability of lockdown and individuals’ perceptions of lockdown across the globe. |
| 2. Secondly, and relatedly, I am concerned about the limited generalizability of the implications of this study. Since lockdowns are a once in a lifetime event as a global pandemic of this scale is unlikely to be frequent, the authors are unable to convey the meaning and implications of the findings to either governments, global development agencies or health systems. What do we take away from this analysis that can enable greater systemic preparedness in the future? And is that preparedness one for governments or systems? | We hope that a lockdown of this scale may be the sole one we experience in our lifetime. However, the head of the WHO Emergencies Program has warned that “the next pandemic may be more severe”. The past twenty years has seen three main pandemics: the 2003 severe acute respiratory syndrome (SARS) pandemic; the 2012 Middle eastern respiratory syndrome (MERS) pandemic; and the ongoing COVID-19 pandemic, all of which have required lockdowns. Of further concern is the resurgence of viral haemorrhagic diseases, such as Ebola, that have caused further local lockdowns. Despite a long history of pandemics that has led to multiple lockdowns/quarantines since the plague of the 14^th^ century, many countries were unprepared for the COVID-19 pandemic. Nor are these issues behind us. China’s ‘zero COVID’ policy continues to impose strict restrictions on its population. As such, our findings have relevance to current as well as future restrictions.  The findings of our review are not restricted to large-scale pandemic lockdowns as they are applicable to both country-wide and local lockdowns across the globe. Our findings indicate that individuals’ perceptions of coercion, pressures and injustice affect their behaviour and, thus, infection control. Individuals overall were accepting of brief and intensive restrictions. Longer, uninterrupted restrictions were less tolerate as viewed as intrusive and, thus, potentially coercive and unjust. As indicated by the literature found in this scoping review, such perceptions are shaped by governmental messaging and the perceptions and behaviour of others which are, in turn, also influenced by governmental messaging. The review is limited by the available research on an ongoing pandemic, however, the included studies give early indications as to how to inform future policy and practice. Pertaining to messaging, as written within the review, the evidence suggests that those who place less trust in authorities may be less likely to respond positively to commanding messages if these are perceived as threatening to their autonomy (ie. perceived as coercive).  Of relevance to health systems’ preparedness, low perceived control was linked to greater depressive and anxious symptomatology. This may translate to a greater need for mental health services. As psychologists based in the UK, we saw an increased waiting time for access to secondary mental health services as colleagues were redeployed during the pandemic. This likely resulted in fewer available resources for those with more complex presentations and less access to support for new referrals with more complex clinical presentations, and a need for contingency plans to be put in place to prepare for future lockdowns. However, we are aware that access to services and health budgets differ between countries and, therefore, we cannot make recommendations that fit all.  Finally, the review also suggests that individuals with fewer economic resources may be disproportionately affected by blanket lockdown policies and that procedurally just policies should aim to provide equitable, rather than equal, support for those who are more socioeconomically deprived. |
| 3. The themes investigated in this review largely focus on trust, pressure and coercion, but these are political constructs rather than investigated in the context of health. How do the authors justify the relevance of this paper to the journal? | We thank the reviewer for allowing us to clarify our stance. The constructs of perceived coercion and pressures originate from psychiatry rather than politics. Perceived coercion, a term borrowed from the mental health literature in relation to mental health hospital admissions, describes the extent to which an individual believes they have choice, autonomy and control over their circumstances (Gardner et al., 1993). Perceived pressures refers to whether an individual experienced inducements, threats or force over their circumstances whilst procedural justice examines whether the individual felt their voice and needs were listened to and respected (Gardner et al., 1993). Terms such as adherence, acceptance, agreement and trust were also included as these informally indicated the presence and/or absence of perceived coercion and pressures within the searched literature. We hope our amendments to the introduction make our intended meaning clearer. |
| 4. “The review identified major gaps in our knowledge pertaining to the absence of information regarding specific individual characteristics and circumstances that increase the likelihood of experiencing perceived coercion and its related constructs. It also highlighted the absence of standardised measures of perceived coercion, pressures and procedural justice that could be adopted globally, and a need for a better understanding of the cultural and socioeconomic factors affecting adherence to lockdown.” My suggestion for the authors is to provide greater clarity in the abstract as to what those gaps are instead of referring to them. Are the measures needed for health systems and where or in what frameworks would these measures be used? | We thank the reviewer for this comment. We are limited by the permitted abstract word count, however, have specified that the measures would be most applicable for research and clinical practice in mental health services. We have also specified that a need for a better understanding of the cultural and socioeconomic factors affecting adherence to lockdown must be underpinned by a need to make socioeconomical support and access to resources equitable. |
