# Supplementary material for: Perceived coercion, perceived pressures and procedural justice arising from global lockdowns during the COVID-19 pandemic: A scoping review
Source: PLOS Glob Public Health. 2023 Mar 16;3(3):e0001250. doi: 10.1371/journal.pgph.0001250 (PMC10019622; doi:10.1371/journal.pgph.0001250)
Supplement: Attachment — Submitted filename: PLOSGPH_Response_to_Reviewers_03.02.23.docx [file pgph.0001250.s003.docx]

| Reviewer 4 comment | Authors’ changes to manuscript |
| --- | --- |
| 1. Abstract: The last 2-3 lines which summarise the key results of the study appear to be overly abstract/satellite view of the studies. While these are acceptable as results, I suggest the authors to re-visit the framing of what the key results of this review are. Currently, both points related to “absence of standardiosed measures” and “better understanding of cultural and socioeconomic factors” are both well known. I believe the authors do have specific review findings that they could report in the abstract. | We thank the reviewer for this comment. We have amended the results and discussion section of the abstract to state the following: *“Our synthesis suggests that i) individuals experienced an initial willingness and tolerance of lockdown that lessened over time as perceptions of personal control decreased; ii) that social influences may pressure individuals to follow or break lockdown rules; and iii) that justifiability and proportionality together with individuals’ perceptions of harm from COVID-19 may impact the extent to which individuals adhere to lockdown. Furthermore, the review found an absence of information regarding specific individual characteristics and circumstances that increase the likelihood of experiencing perceived coercion and its related constructs and highlights a need for a better understanding of the cultural and socioeconomic factors affecting perceptions of, and adherence to, lockdown.”* |
| 2. The track-changes document contains unresolved commentary between co-authors that should ideally have been resolved before submission. | We apologise for this error and have rectified it. |
| 3. On the issue highlighted above about limited regional/subregional/  ethnic/indigenous/socio-demographic patterning of results at smaller scales, see  for eg. sections in the Results (esp. theme 1) where assertions are made at global level.  These require to be more modestly framed and attributed to specific populations/regions/demographies based on the study they are coming from. For instance,  (a) on p.9 “most participants expressed willingness to…” (begging the question where? And which kind of participants?), (b) on p.23 “…correlated with belief that political leaders are competent….(where was this study because perceptions of trust on elected representatives or political institutions is widely variable even within countries), (c) on p.23 again “…some studies suggested that participants from economically developing countries” (aside from the fact that the category economically developing countries is problematic, it is unclear which kind of participants/people held this perception; where they too from HIC/LIC/LMIC), (d) “individuals with right wing political leanings…” (given that what is considered “right wing” is differently perceived and understood both in global health and within/across countries, this and other findings like this will need a bit more contextualisation), (e) on p.24, “similarly low-income participants were less in favour of lockdown” (again, where?); also see later on same page reporting findings which need regionalisation/contextualisation, (f) on p.28, see “Those opposed to lockdown tended to express greater distrust in authorities” (again this is not a globalisable finding no? so where was this or else in what kind of settings was this this the case?) | We thank the reviewer for this comment. We have amended the results section so that it is more specific and culturally sensitive. |
| 4. On p.4, fix the sentence synax/grammar: “The purpose of this review is…” | We thank the reviewer for pointing this out. That sentence has now been amended to: *“The purpose of this review is to inform both our understanding of and public health policies on the factors that contribute to greater perceived coercion, with a view to comprehending how such factors may impact on psychological wellbeing and other affiliated factors.”* |
| 5. The authors (on p.5) assert their intention to share “gaps in knowledge” and later on have an entire section on p.32 on “Areas for future research”. I find this challenging to review. Methodologically speaking, how were these gaps identified? Was an a priori framework used to identify this? For instance, based on my reading of the manuscript, I can identify various other gaps and this depends on the position/framework of the reader. In the lack of that, it is tough to critically assess if the gap identification was based on the evidence reviewed (or not). I would suggest to the authors to moderate/drop this objective as well as the section on p.32 on areas for future research as these appear to lack substantial justification in the methods. Even without these, the article still has important  contributions. | We thank the reviewer for this comment. We have now omitted those sections. |
| 6. There have been extensive commentary on lockdowns and their perceived effects in grey  literature and in-depth analysis of various narratives on these. Some of these narratives go far beyond descriptive journalistic pursuits to more deeply inquire into the diversity of  lockdown impacts faced by several vulnerable groups. The review’s lack of engagement with this must be acknowledged as a limitation. | We were unable to add such literature due to having limited manpower within a small research team. We have included a sentence within the strengths and limitations section of the discussion stating the following: “*Missing from this picture is also an account of the grey literature on the topic.”* |
| 7. On p.6, the authors have now added a few lines (in track-changes) starting with  “Examples of excluded search terms were….”. This is a bit puzzling because many of these were part of the search strategy. Please review this portion carefully once more. | This is correct. Though many of the words within the search terms are the same, the amalgamation of these are different. The search term: *(adherence OR acceptance OR agreement OR trust OR distrust OR compliance OR willing*) OR (perceived coerc* OR percept* coerc* OR pressure OR force OR influence OR control OR threat OR justice) AND (quarantine OR lockdown OR isolation) w*as excluded as it did not pertain specifically to COVID-19 and was overly sensitive.  The search term: (*perceived coerc* OR percept* coerc* OR pressure OR force OR influence OR control OR threat OR justice) AND (COVID-19 or coronavirus)* was excluded as it was not sensitive enough.  The term we found optimal was *(adherence OR acceptance OR agreement OR trust OR distrust OR compliance OR willing*) OR (perceived coerc* OR percept* coerc* OR pressure OR force OR influence OR control OR threat OR justice) AND (lockdown) AND (COVID OR SARS-CoV-2 OR COVID-19).* |
| 8. On. p.25, use of the word “chronicled” may be reviewed. One of the papers attributed to  be such a “chronicle” is a qualitative study of predictors. | The word “chronicled” has been amended to “recorded”. |
| 9. Review the bringing up of a systematic review as a citation (on p.24). IS this the only one on this topic? | We thank the reviewer for this comment. This is the only systematic review on entrapment/perceived control that emerged from our database search. |
| 10. “Conspiracy theory” is brought up based on its study from 1 of the papers authors  reviewed. This comes back again in the Discussion where there is reference to Whatsapp messaging and specific political leaders (Trump for example). I am not too convinced on the way in which this topic has been handled as there is very little empirical basis presented by the authors to be able to make these assertions in the results and discuss. Please review this critically. | As the topic of conspiracy theories emerged from the included studies, we have included this within the results. However, we have omitted reflections on this within the discussion so as to not place an emphasis on the topic in light of a need for further evidence and potential inapplicability to non-Western cultures. |
| 11. One of the issues is that phenomena such as “collective social norms” (which appears on p.26 in the context of a study from Indonesia and Malaysia) is one of several phenomena of interest that the authors bring up in their review which is highly contextual, socio-cultural and political and requires in-depth and contextualised inquiry. However, this has not been substantively discussed and is often presented in a globalised way. For eg. the collectivistic social norms that were important enough to emerge from the study they reviewed from Indonesia and Malaysia may not at all be powerful enough to explain similar outcomes, say in Morocco or in UK. How do we then moderate our assertions to be able to convey such a nuance? (see point 3) | We thank the reviewer for this comment. As psychologists and ethicists, we feel it is best for us to urge caution to the readers regarding the generalisability of this finding rather than discuss the contextuality of it. Therefore, *w*e have included the following sentence within the results to reflect that the studies pointing to collective social norms are few in number: *“Societal norms were also reported to play a role in individuals’ perceptions regarding lockdown in a minority of studies”* The following sentence has also been included within the discussion: *“However, as the studies pertaining to social norms are few in number, these may not be generalisable to other countries.”* |
| 12. Some of the topics that come up in discussion were outside the remit of the review. Please carefully review the Discussion to remove these. For instance, see the section on p.31 on “what level of restriction is justified for what level of risk of harm….” | We politely disagree with this comment. Individuals’ justification of lockdown is likely to affect their perception of coercion arising from such restrictions. Such perceptions and justifications are likely impacted by the presence or absence of socioeconomic privilege as well as perceptions of risk. |
